# Composition, Optical Resonances, and Doping of InP/InGaP Nanowires for Tandem Solar Cells: a Micro-Raman Analysis

**DOI:** 10.1021/acsnano.3c12973

**Published:** 2024-03-27

**Authors:** Irene Mediavilla, Jose Luis Pura, Vanessa Giselle Hinojosa, Beatriz Galiana, Lukas Hrachowina, Magnus T. Borgström, Juan Jimenez

**Affiliations:** † GdS Optronlab, Ed. LUCIA, Universidad de Valladolid, Paseo de Belen 19, 47011 Valladolid, Spain; ‡ Instituto de Estructura de la Materia (IEM-CSIC), Consejo Superior de Investigaciones Científicas, Serrano 121, 28006 Madrid, Spain; § Universidad Carlos III de Madrid, Physics Department, Av. Universidad 40, Leganes 28911, Spain; ∥ Nano Lund and Division of Solid State Physics, Lund University, Box 118, 22100 Lund, Sweden

**Keywords:** semiconductor nanowires, Raman, tunnel diode, plasmon modes, InGaP, InP axial heterostructures, alloy composition

## Abstract

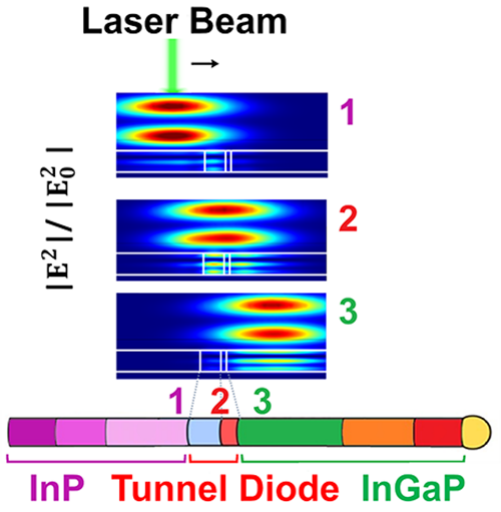

We present a micro-Raman study of InP/InGaP tandem junction photovoltaic nanowires. These nanowires render possible InGaP compositions that cannot be made in thin films due to strain. The micro-Raman spectra acquired along the nanowires reveal the existence of compositional changes in the InGaP alloy associated with the doping sequence. The heavily Zn-doped In_*x*_Ga_1–*x*_P (*x* is the In molar fraction) side of the tunnel diode is Ga rich, *x* = 0.25, with respect to the n-type and intrinsic segments of the top cell, which are close to the nominal composition of the NWs (*x* = 0.35). The p-type end segment is still Ga-rich. Electromagnetic resonances are observed in the tunnel diode. The Raman signal arising from the InGaP side of the tunnel diode is significantly enhanced. This enhancement permits the observation of a Raman mode that can be associated with an LO phonon plasmon coupled mode (LOPCM). This mode has not been previously reported in the literature of InGaP, and it permits the Raman characterization of the tunnel diode. The analysis of this mode and its relation to the LO phonon modes of the alloy, InP-like and GaP-like, allows to establish an apparent one-mode behavior for the phonon plasmon coupling. It indicates that hole plasma couples to the GaP-like LO mode. The LOPCMs are modeled using the Lindhard Mermin formalism for the dielectric function.

Semiconductor nanowires (NWs) constitute a technological platform for the next generation of advanced nanodevices for photonics, optoelectronics, and sensors, among others.^1^ NWs permit the assembly of complex structures with lattice mismatched materials not feasible in a planar configuration. Thus, one can combine materials with different bandgaps to form heterojunctions unachievable in the planar technologies allowing to setting up nanodevices with improved functionalities, e.g., NWs with optimized absorption of the solar spectrum for high-efficiency solar cells.^2^ Subwavelength diameter NWs behave like optical nanoantennas,^3^ which makes them suitable for a broad range of applications in nanophotonics,^4,5^ highly efficient light sensors,^6^ and solar cells.^2^ Their optical properties can be tuned through the shape (diameter and length), the dielectric mismatch with the environment, and the use of heterostructured NW synthesis. The NWs can trap much more light per volume unit than planar structures, which makes them suitable for sustainable photon harvesting with respect to material consumption.^7^ Light absorption can be tailored by the NW geometry, structure, and material composition. Doping, the formation of both homo- and heterojunctions, and the NW morphology and composition control are crucial issues for achieving NW devices.

The growth of NWs by use of the vapor liquid solid (VLS) growth mode presents several challenges related to reservoir effects of materials soluble in the catalyst droplets, which affect composition control, incorporation of dopants, abruptness of the junctions, and diameter control.^8−11^ Furthermore, one has to inhibit parasitic radial growth which results in a characteristic tapered shape of low-quality material on the NWs walls.^12^ Nanoscale analysis is crucial to probe individual NWs with complex structures in order to assess composition fluctuations, doping characteristics, and material properties.

The enhanced optical absorption of NWs permits to characterize single ones by micro-Raman spectroscopy, reaching a Raman signal roughly equivalent to the signal obtained in bulk material under the same experimental conditions in spite of its reduced scattering volume.^13^ There is extensive literature about the characterization of semiconductor NWs by using micro-Raman spectroscopy. The Raman spectrum provides information about different fundamental properties of NWs, namely, one can determine the composition in alloyed NWs,^14^ strain,^15^ thermal transport,^16,17^ crystal phases,^18^ and characterization of the heterojunctions (HJs) through optical resonances.^19^ In addition, the free carrier concentration and mobility can be estimated through the LO phonon plasmon coupled modes (LOPCMs) in polar semiconductor NWs.^20^ This is particularly useful because the method does not require the challenging formation of transparent nanosized electrical contacts. A critical point concerns the abruptness of the HJs, which in axial HJs can result in composition gradients extending up to a few tens of nanometers depending on the growth conditions and the solubility in the catalyst droplets of the elements forming the NW.^8^ In particular, tunnel diodes demand a sharp interface with a very narrow space charge region to allow tunneling.^21^

In_*x*_Ga_1–*x*_P has a composition-dependent bandgap and is used, for instance, in the world record tandem junction solar cells.^22^ Tandem solar cells are formed by connecting several subcells in series, separated by tunnel diodes that ensure carrier transport between subcells. The tunnel diode must be degenerately doped to warrant a narrow depletion width, which is challenging to achieve with NWs, despite which NW tunnel diodes (In_*x*_Ga_1–*x*_P/InP) were demonstrated.^21^

We present a micro-Raman study of NWs with a tandem solar cell structure consisting of a n-i-p InP bottom cell and a n-i-p InGaP top cell connected by a p^+^-In_*x*_Ga_1–*x*_P/n^+^-InP tunnel diode (see Figure 1). We analyze the influence of doping on the composition along the different sectors of the NWs, the optical resonances, and the interaction between the free carrier plasma and the longitudinal optic phonons, which evidence an apparent one-mode (GaP-like) behavior for the LOPCMs.

**Figure 1:**
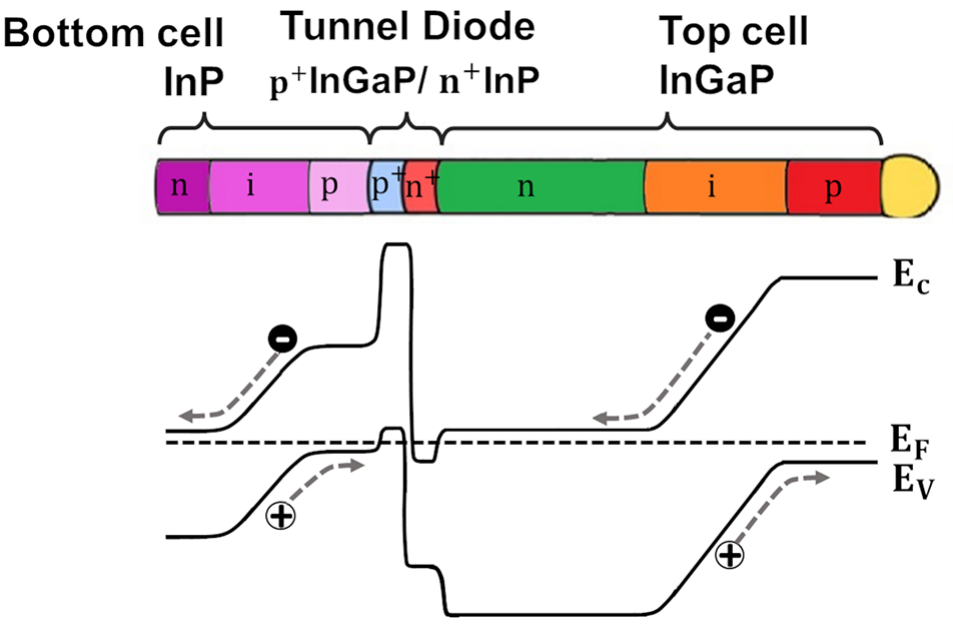
Schematic of the heterostructured NW and bandgap diagram.

## Raman Spectroscopy of InGaP

The assignment of the Raman bands in In_*x*_Ga_1–*x*_P is a matter of controversy.^23^ A typical Raman spectrum of a (100) In_*x*_Ga_1–*x*_P layer presents three main spectral features: (i) a GaP-like LO peak, henceforth labeled LO1, with a frequency ranging from 348 cm^–1^ (*x* = 1) to 405 cm^–1^ (*x* = 0), (ii) A TO peak, henceforth TOM, with frequency ranging from the TO InP frequency, 304 cm^–1^ (*x* = 1), to the TO GaP frequency, 368 cm^–1^ (*x* = 0), and (iii) a peak with frequency ranging from 345 cm^–1^ (*x* = 1) to 395 cm^–1^ (*x* = 0). In the one-mode approach, this last peak is associated with a disorder-activated mode,^24^ while in the two-mode scenario, it is associated with the InP-like LO mode, henceforth labeled LO2.^25,26^ The observation of the TO mode in this scattering configuration is due to alloy disorder. In the modified two-mode behavior, there are two LO phonon modes, namely, InP-like (LO2) and GaP-like (LO1), while the TO phonons obey one mode behavior.^27,28^ In the two-mode scenario, another peak with frequency between the two LO modes has been associated with a TO phonon, labeled TOm. The mode strength of the low-frequency TO phonon (TOM) is at least 1 order of magnitude higher than that of the high-frequency TO phonon (TOm),^29^ furthermore, this last mode has been scarcely reported, because it is hidden by the LO modes. It was identified as a pure GaP mode instead of as the InP-GaP mixed TOM mode.^30,31^ The empirical dependence of the four phonon peak frequencies with the In molar fraction, *x*, is given by^25,32,33^
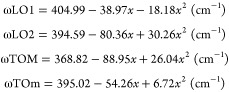
1

## Results and Discussion

The Raman spectra were recorded along the axis of several NWs, characterized by different lengths. Note that all the NWs studied were grown in the same growth run.^34^ Depending on the location on the substrate the growth rate can change, resulting in faster growth in the periphery of the substrate, giving longer NWs.^34^ This can result in compositional and doping differences, as revealed by the corresponding Raman spectra. Long NWs mainly grow at the substrate edge, while short NWs are grown in most parts of the substrate, which are not influenced by the diffusion of precursor materials from the substrate edge.

The results obtained for all NWs show similar trends. The spectra recorded on different NWs are reported in the Supporting Information (SI), section 1 (SI 1). We present here a detailed Raman analysis of a selected NW that is 10 μm long. This length allows better resolution of the different sections of the NW for a clear observation of their corresponding Raman signatures as compared to shorter NWs. In short NWs the contribution of different segments appears mixed in the spectra due to the size of the laser beam diameter (*D*), nominally 0.7 μm, according to the Abbe formula, *D* = 1.22λ/NA (λ = 532 nm, numerical aperture of the objective NA = 0.9). The main Raman features can be observed in all NWs measured, SI 1, but the laser beam size mixes different NW sectors in the short, 2–3 μm, NWs. Therefore, long NWs are suited for better resolution of the different NW sectors. The spectra of the NWs with different lengths permit us to extrapolate the results obtained in the long NWs to those in the shorter NWs.

**Figure 2:**
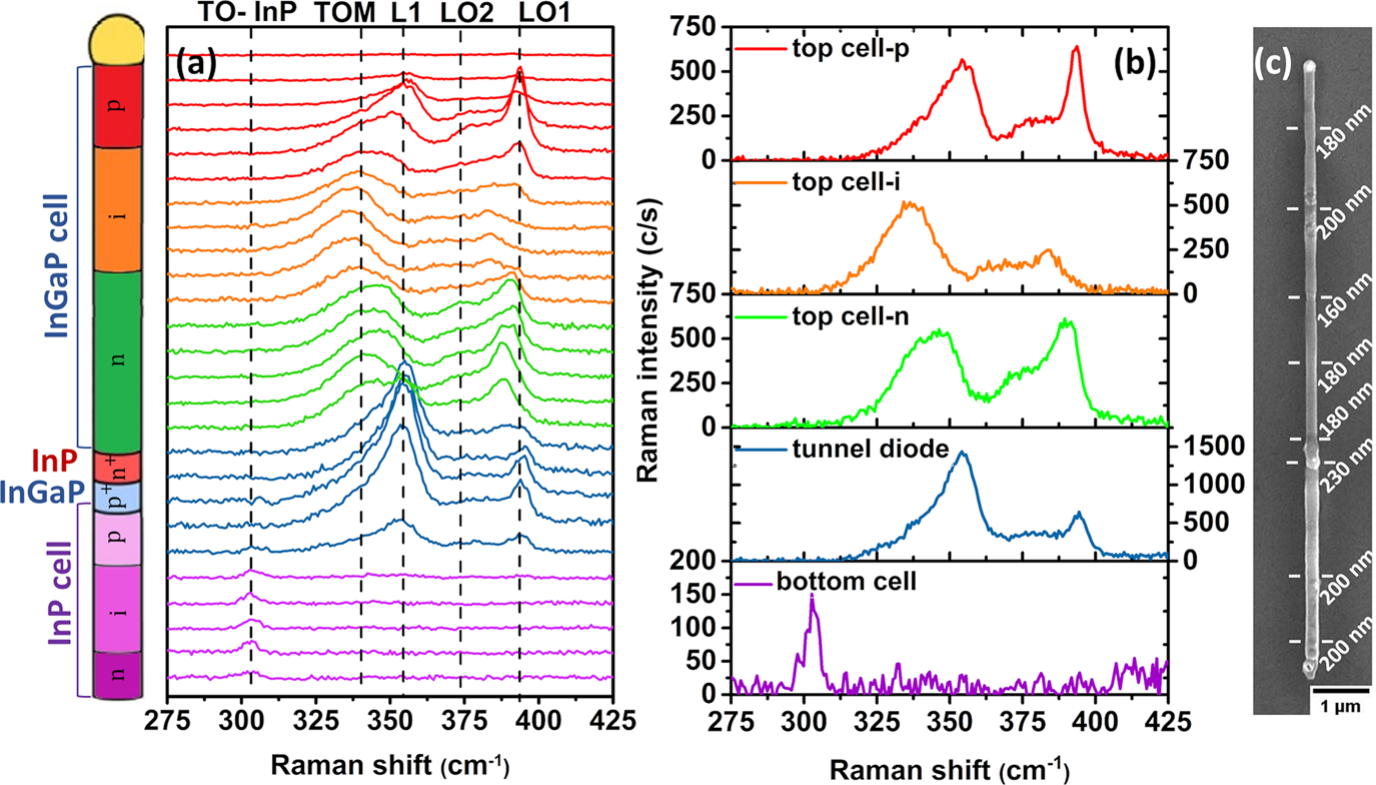
(a) Raman spectra recorded along the NW in steps of 200 nm. (b) Selected Raman spectra representative of the different NW sectors, note the different scales showing the high intensity in the tunnel diode. (c) SEM image of the NW indicating the diameter of the different NW sectors.

The sequence of spectra recorded along the NW is shown in Figure 2. (a) Raman spectra recorded along the NW in steps of 200 nm; (b) Selected Raman spectra representative of the different NW sectors, note the different scales showing the high intensity in the tunnel diode; (c) SEM image of the NW indicating the diameter of the different NW sectors.The diameter is not homogeneous along the NW, but it presents changes associated with the composition and doping sequence. The NW diameter is larger in the bottom cell (>200 nm) than in the top cell where it is about 180 nm.

For a better understanding of the results, the phonon peak frequencies of InP and GaP for both zinc blende (ZB) and wurtzite (WZ) crystal phases are shown in Table 1. Clear differences can be observed between the spectra of the two crystal phases, which facilitates their identification from the Raman spectrum. Also, the peak frequencies of the In_*x*_Ga_1–*x*_P alloy for *x* = 0.25 and 0.35 corresponding to the tunnel diode and the top cell, respectively, are included in the table.

**Table 1:** Raman Modes of InP, GaP in ZB,^36,37,40,41^ and WZ^38,39,42^ Phases and In_*x*_Ga_1–*x*_P ZB (*x* = 0.25 and 0.35) Calculated from Equation 1

	*E*_2_^*L*^ (cm^–1^)	*E*_2_^*H*^ (cm^–1^)	*A*_1_(TO) (cm^–1^)	*E*_1_(TO) (cm^–1^)	*E*_1_(LO) (cm^–1^)	*A*_1_(LO) (cm^–1^)
InP-ZB^36,37^				303.7		341.7
InP-WZ^38,39^		306.4	302.1	302.4	347	341.9
GaP-ZB^40,41^				356		392
GaP-WZ^42^	78.1	355.8	363.4	363.4	401.4	395.7

	TOM (cm^–1^)	LO1 (cm^–1^)	LO2 (cm^–1^)
In_*x*_Ga_1–*x*_P (*x* ≈ 0.25)^25,32,33^	348.2	394.1	376.4
In_*x*_Ga_1–*x*_P (*x* ≈ 0.35)^25,32,33^	340.9	389.1	370.2

The spectra recorded on the InP bottom cell were noisy, exhibiting only a weak peak at ≈304 cm^–1^, corresponding to the TO mode of ZB InP (see Figure 2b). The LO phonon mode could not be observed. This points to a ZB structure and dominant backscattering on the (110) face of the NW, for which the TO mode is allowed, while the LO mode is forbidden. The weak Raman signal of the InP bottom cell is related to the low Raman response of InP and the absence of a diameter resonance of the InP NW segment for 532 nm light excitation. We did not observe wurtzite-like modes (see Table 1), which suggests that this phase is not present in the NW, or that its volume, if any, is insufficient to be detected in the Raman spectrum. Wurtzite phase is more common in NWs with diameters below 25 nm.^18^ Furthermore, Zn doping results in zinc blende InP NWs. The absence of the LO phonon mode rules out the formation of twining superlattices (zinc blende/wurtzite), for which the NW side facets of p-type InP are made up of oscillating (111)B and (111)A morphologies.^35^ In order to confirm the ZB structure of the NW, we carried out high-resolution transmission electron microscopy (HRTEM) measurements. The fast Fourier transforms (FFT) of the TEM images reveal unambiguously the ZB structure of the three main sectors of the NWS, e.g., the bottom cell, the tunnel diode, and the top cell, confirming the Raman results, see SI 2.

When the laser beam enters the tunnel diode, the Raman signal arising from the p^+^-InGaP side of the tunnel diode is enhanced. The Raman spectrum of the tunnel diode exhibits three main peaks, i.e., an asymmetric band with a high intensity peaking at ≈355 cm^–1^ (labeled L1), largely more intense than all the other Raman bands collected all along the NW and the two LO peaks of InGaP at ≈394 cm^–1^ (LO1) and ≈377 cm^–1^ (LO2), respectively. Following the symmetry observed in the InP bottom cell, these LO phonons should be forbidden; however, the observation of LO modes points to a breakdown of the Raman selection rule in the tunnel diode. The high intensity of the L1 peak accounts for strong electromagnetic resonance at the p^+^-InGaP side of the tunnel diode. The Raman intensity in the tunnel diode has to be weighted by the scattering volume and the laser intensity distribution. The Gaussian laser beam intensity distribution in micro-Raman measurements needs to be taken into account for the intensity analysis. According to the Abbe formula, the laser beam diameter at focus is ≈0.7 μm. The tunnel diode is a thin slab (≈ 200 nm); therefore, the scattering volume corresponding to the p^+^-InGaP side of the tunnel diode is nearly 25% of the scattering volume probed in a homogeneous NW segment longer than the laser beam diameter. Therefore, the intensity of the spectra arising from the tunnel diode has to be enhanced by a factor of ≈4 to be compared to the spectra recorded on the other sectors of the NW. This intensity increase should give the real figures of the electromagnetic field enhancement at the tunnel diode and more in particular at its p^+^-InGaP side.

**Figure 3:**
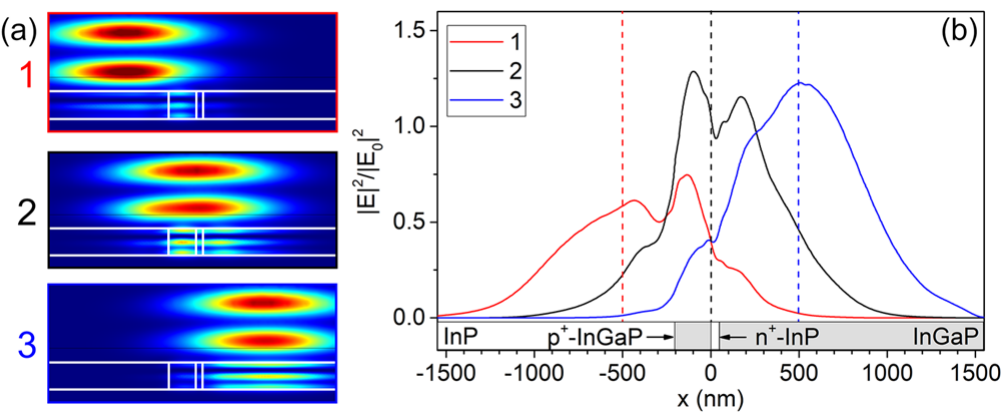
(a) Simulation of the electromagnetic field distribution when the laser beam is focused on different positions around the tunnel diode indicated by the dashed lines: (1) on the p^+^-InGaP side of the tunnel diode, (2) on the n^+^-InP side of the tunnel diode, and (3) on n-InGaP side of the top cell. (b) The intensity profiles along the NW axis, |*E*|^2^/|*E*_0_|^2^, for the three laser beam positions: red (1), black (2), and blue (3).

The asymmetric shape of the L1 band is the consequence of a residual TOM contribution that stretches out the low-frequency side of the band. It should be noted that the n^+^-InP side of the tunnel diode, with a length of 30–50 nm depending on the NW length, is silent in the Raman spectrum. The enhanced optical absorption at NW axial HJs was studied in previous articles.^13,43^ It was shown that strong local optical resonances can take place at the HJ, mainly on one side of the HJ depending on the refractive index mismatch between the two sides of the HJ.^44^Figure 3a shows the calculated electromagnetic field distribution inside the NW under the excitation with the laser beam. The electromagnetic field distribution was calculated by using COMSOL multiphysics. The geometric parameters are taken from the SEM image to simulate the actual NW dimension, Figure 2c. The dielectric properties of each segment are fixed in agreement with the literature.^45^ The system is illuminated by a 532 nm Gaussian beam, replicating the laser excitation of the Raman spectra. The simulations provide the normalized distribution of the electric field intensity inside the NW, |E|^2^/|E_0_|^2^, for different positions of the excitation beam, as shown in Figure 3a. The Raman signal generated in each segment will be proportional to this magnitude, and it shall depend on the laser beam position. The electromagnetic field is enhanced at both sides of the n^+^-InP segment of the tunnel diode, and it is quenched inside the n^+^-InP section, impeding the observation of the Raman spectrum arising from it, see the profiles of |*E*|^2^/|*E*_0_|^2^ along the NW axis for the three laser positions, Figure 3b. The dielectric functions used for this calculation take account of both the phonon and free carrier contributions using the Lindhard–Mermin formalism described in the Supporting Information (SI 3).

When the laser beam enters the top cell, starting with the n-InGaP segment, one observes three bands that can be associated with TOM, LO2, and LO1 phonons. The LO1 band is broadened with respect to that of undoped InGaP, which is a consequence of n-doping. It is worth mentioning that the TOM peak presents a shoulder on its high-frequency side. We will return to this later on. In the i-InGaP intrinsic segment of the top cell, one observes the TOM band, which is dominant along this segment over the very weak LO bands, which matches the Raman selection rules for backscattering on the (110) faces of the ZB phase, as observed in the InP bottom cell. In the end segment of the top cell, p-InGaP, one observes an enhancement of the Raman signal, which is less significant than that observed in the tunnel diode. In this region, approaching the catalyst gold droplet, the L1 band reappears. The LO1/L1 intensity ratio is much higher than that in the tunnel diode. This might be related to the lower free hole concentration in this section as compared to the degenerately doped tunnel diode. A Raman resonance effect at the interface with the gold catalyst droplet was reported elsewhere.^14^ The Raman selection rule breakdown observed in the InGaP doped segments, both n and p, suggests that forbidden modes are activated as a consequence of the breakdown of the phonon wave-vector conservation rule, because of phonon scattering by ionized impurities.^46^ Furthermore, the breakdown of the symmetry selection rule close to the HJs of the tunnel diode and nearby the gold droplet can be partly contributed by the sharp electromagnetic field gradient associated with local optical resonances.^47^ Note that the Raman intensity at the tip was reduced in NWs without gold droplets as compared to the signal recorded in NWs with the gold droplet remaining on top; see SI 1.

**Figure 4:**
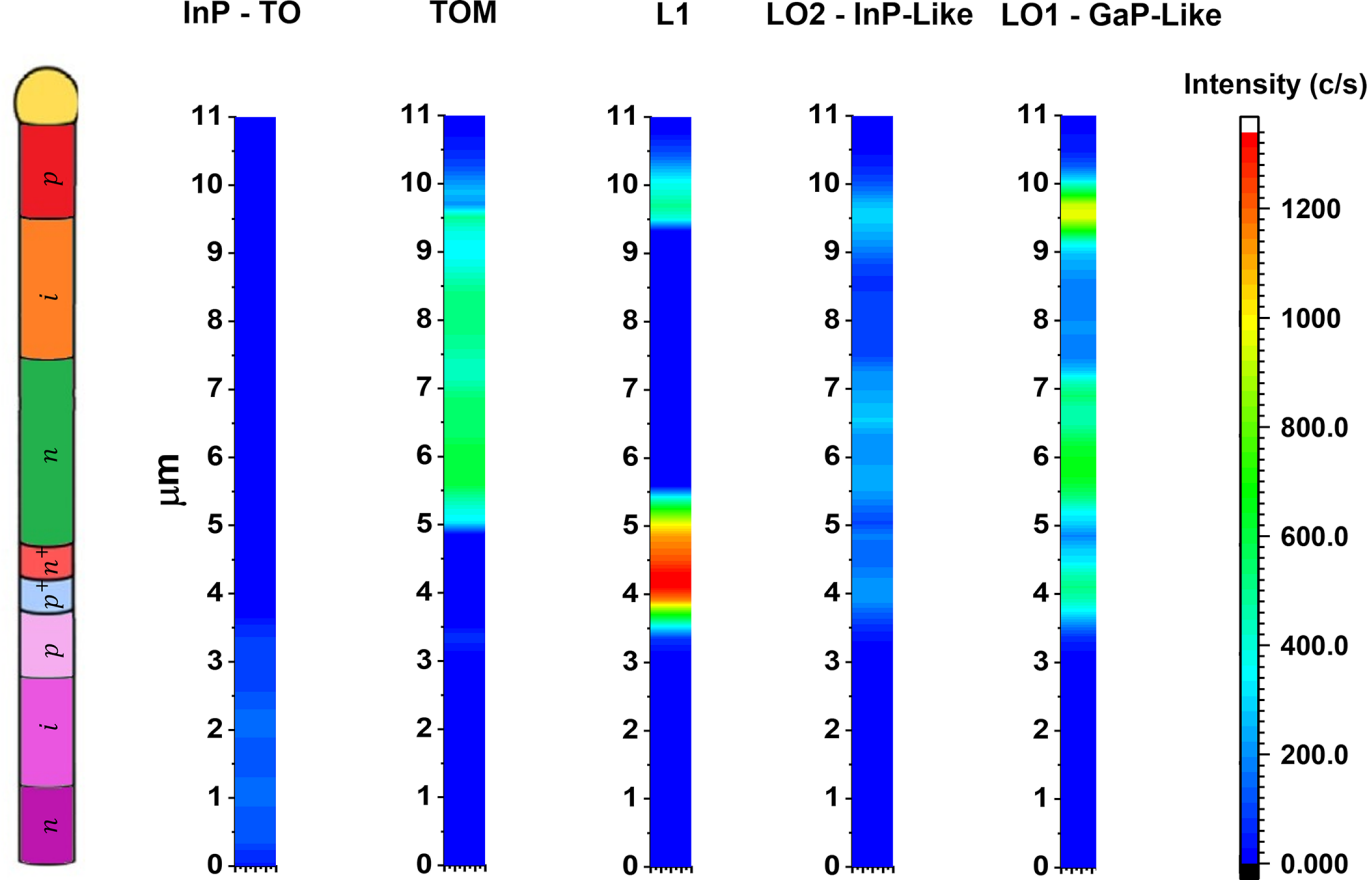
Distribution of the different Raman bands along the NW, as obtained by the spectra fitting by Gauss/Lorentz functions.

The analysis of the spectra recorded by the laser beam scanning permits us to visualize the distribution of the different Raman bands along the NW, Figure 4.

The L1 band is observed in the p-doped segments (tunnel diode and NW tip). While the TOM mode is mainly observed in the n-InGaP, and i-InGaP segments of the top cell. The LO bands are observed in the tunnel diode and in the doped segments of the top cell, n-InGaP, and p-InGaP, while it is almost negligible in the i-InGaP segment, its presence denotes Raman selection rule breakdown as compared to the bottom cell and the intrinsic InGaP segment of the top cell. The intensity of the different bands highlights the resonance effect in the p^+^-InGaP side of the tunnel diode. The structure is still ZB, as seen by HRTEM (SI 2).

Because the Raman shift of the LO1 phonon is almost not affected by the spontaneous alloy ordering,^48,49^ it can be used to estimate the composition of the different segments of the NW in the absence of strain.^32,50^

**Figure 5:**
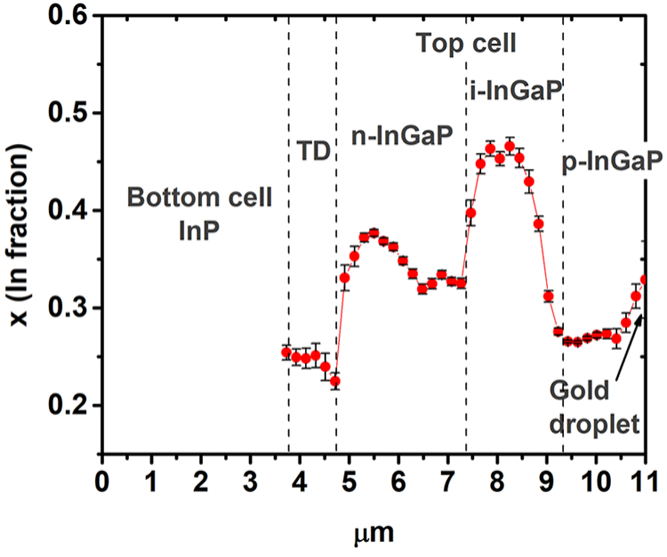
In fraction distribution along the NW was obtained from the LO1 peak frequency (eq 1).

The calculated value of *x* along the NW using eq 1 is plotted in Figure 5. These values are in good agreement with energy-dispersive X-ray spectroscopy (EDX) measurements (see SI 4). It appears that Zn doping reduces substantially the incorporation of indium, giving a Ga-rich alloy in both the tunnel diode (*x* ≈ 0.25) and the p side of the top cell (*x* ≈ 0.25–0.3). This is due to the use of diethyl-zinc (DEZn) during growth, which facilitates the incorporation of gallium.^51^ Doping with S does not introduce significant changes in the In incorporation (*x* ≈ 0.35). The nominally intrinsic In_*x*_Ga_1–*x*_P segment has *x* ≈ 0.35, instead of the *x* ≈ 0.4 estimated from the LO1 peak, although the Raman assignment in the i-InGaP segment presents the highest uncertainty because of the very weak LO1 phonon band recorded in this segment. The dashed lines delimiting the different segments in Figure 5 are only approximate, since the size of the laser beam does not allow a precise separation between the different sectors.

We previously identified the phonon bands of InGaP, which are observed in different zones of the tunnel junction and the top cell. However, the L1 band observed in the p-doped sections of the NW does not match any of the characteristic bands of InGaP. Interesting characteristics of this band are (1) it is observed in the p-doped InGaP sections of the NW, (2) its intensity is higher in the p^+^-InGaP side of the tunnel diode than in the p-InGaP side of the top cell, close to the catalysts gold droplet, where the free hole concentration is lower, and (3) it follows the same symmetry rule as the LO phonons, and the higher its intensity the lower the LO1 band intensity.

**Figure 6:**
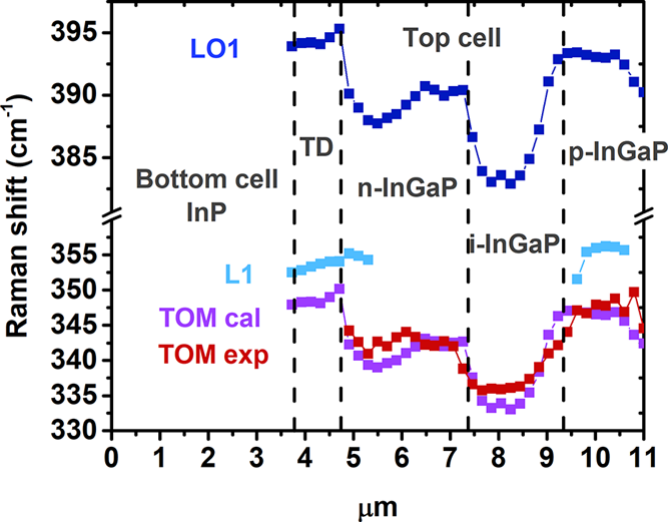
Raman peak frequencies of LO1, L1, and TOM along the NW. TOM frequency calculated for the composition estimated from the LO1 peak frequency using eq 1 is also plotted.

We focus on the frequencies of the different Raman bands. The peak frequencies of the LO1 and TOM bands are experimentally measured. The peak frequency of the TOM band is calculated according to eq 1 for the alloy composition, which is deduced from the experimental LO1 peak frequency. The LO1, experimental TOM, calculated TOM, and L1 peak frequencies are plotted along the NW length in Figure 6.

The experimental TOM frequency matches the calculated one using the values of x obtained from the LO1 peak frequency, Figure 6. This frequency matching permits to assume that the peak frequencies measured in the InGaP NWs are similar to those measured in strain-free layers.^32,33^ Interestingly, the L1 band peaks at a frequency higher than that of the calculated TOM band. Therefore, one cannot identify it with the TOM band. Since the L1 band is observed in the p-type InGaP segments, one can argue that it is related to the p-doping. We tentatively associate it with an LO phonon–plasmon coupled mode (LOPCM).

## Phonon–Plasmon Coupled Modes

**Figure 7:**
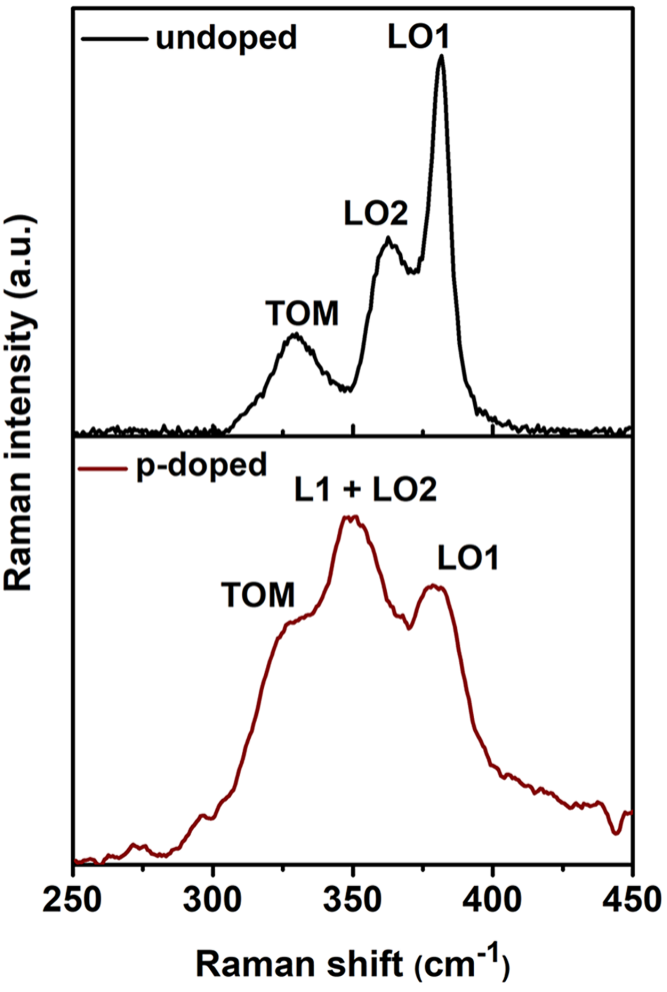
Raman spectra of In_0.5_Ga_0.5_P layers, undoped (top panel) and Zn-doped (lower panel).

In polar semiconductors, the interaction between LO phonons and plasmon modes through their associated macroscopic electric fields results in the formation of LOPCMs. There is exhaustive literature about the LOPCMs in binary III–V compounds,^52−55^ but much less about their alloys and even less in p-doped alloys. In particular, to the best of our knowledge, there is no literature about LOPCMs in p-doped InGaP. In overdamped plasmas, only one LOPCM has been reported in the range of the optical phonon frequencies^54,55^ instead of the two LOPCM branches observed in low-damped plasmas.^51^ In order to get insight about the origin of the so-called L1 band, we performed Raman measurements on Zn doped InGaP epitaxial layers nominally lattice-matched to (100) GaAs substrate, In_0.5_Ga_0.5_P, with a hole concentration ≈4 × 10^18^ cm^–3^, as deduced from Hall effect measurements, Figure 7.

**Figure 8:**
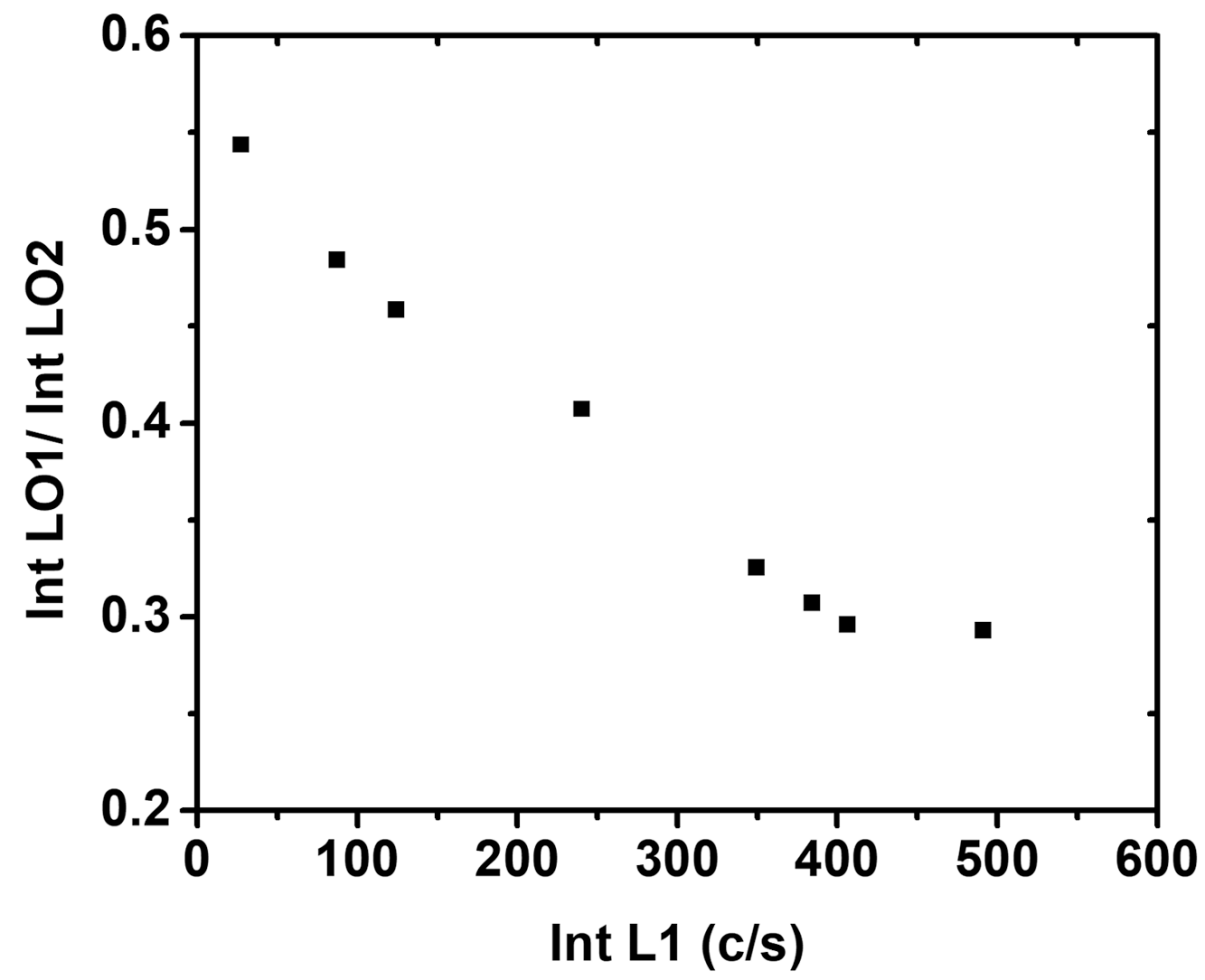
Relative intensity LO1/LO2 vs intensity of L1.

The spectrum of the undoped sample, used as a reference, exhibits the typical spectrum of (100) In_0.5_Ga_0.5_P, with the LO1, LO2, and TOM bands. The LO1 band gives the most intense Raman signal. The spectrum of the p-doped sample shows significant differences with the undoped sample; namely, the intensity of the LO1 peak becomes less intense than a broad band including the LO2 peak and a new peak at 345 cm^–1^. We attribute the changes in the Raman spectrum from the p-type samples to the interaction between LO phonons and free holes. Upon fitting the spectra, one obtains a significant decrease of the LO1/LO2 intensity ratio in the p-doped sample, suggesting that the hole plasma oscillations mainly couple to the LO1 phonon mode of InGaP. The few existing articles regarding Raman characterization of n-type doped InGaP suggest preferential coupling of the electron plasma oscillations to LO1 phonons.^29,44^ Peak L1, recorded from the p-doped segments of the NW is in agreement with the data in references.^29,44^ It has the symmetry of the LO phonon and appears between the LO1 and TOM peaks. It appears closer to the TOM peak than in the epitaxial layers probably because of the higher hole concentration in the NWs. The plasmon–LO coupling screens the LO phonon, whose intensity should decrease with increasing free hole concentration as the surface carrier depletion depth becomes thinner; the LO1 phonon band arises from the carrier-depleted region. The relative intensity of LO1/LO2 vs the L1 intensity obtained for different spectra recorded in the p-InGaP segments of the NWs is plotted in Figure 8, showing a decrease of the LO1/LO2 ratio with increasing L1 intensity. This supports the preferential coupling of the LO1 phonon mode to the hole plasma. On the other hand, the peak frequency of the L1 band is found to be almost insensitive to the free hole concentration, which can be due to strong plasma damping, mainly involving the heavy hole plasma.^56,57^ It has previously been claimed that a one-mode behavior fits the phonon–plasmon coupling in n-doped InGaP better than a two-mode behavior.^29^ We associate this one mode behavior with the dominant GaP-like optical phonon (LO1) over the InP-like optical phonon (LO2). The larger activity of the GaP-like band, LO1, has previously been attributed to charge transfer toward the GaP bonds.^58^ This charge transfer is due to the large bond length mismatch between the two sublattices,^59^ and it would be responsible for the apparent one-mode behavior observed in the LOPCMs.^29^

**Figure 9:**
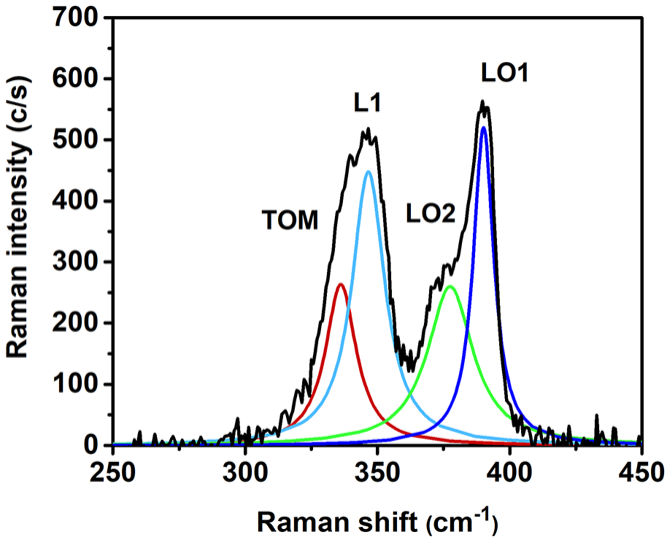
Raman spectrum of the n-InGaP NW sector showing an L1-like peak close to the TOM phonon peak.

The n-InGaP segment composition (*x* ≈ 0.35) corresponds to a band gap close to the direct to indirect bandgap crossover; therefore, the X valley is only a few *k*_B_*T*’s energy above the Γ valley. Therefore, the X valley can be thermally populated at room temperature, resulting in a two-component plasma. The heavier effective mass of electrons in the X valley results in a damped electron plasma^57^ with the consequence of an LOPCM inside the LO1-TOM optical gap of InGaP. In fact, a shoulder on the high-frequency side of the TOM band is observed in the spectrum of the n-InGaP segment, Figure 9. This can be associated with a band, reported by Sinha et al.^23^ and Lee et al.,^29^ appearing in the high frequency side of the TOM band in n-type InGaP.

Alternatively, a narrow Raman band peaking at 354 cm^–1^ in In_*x*_Ga_1–*x*_P layers lattice matched to GaAs (*x* ≈ 0.5) has been reported.^60,61^ It was observed in Cu–Pt like ordered alloys,^62^ and its intensity was found to increase with the degree of order. It was attributed to either an uncoupled plasmon,^60^ or an InP-like LO phonon of mixed *A*_1_–*E* symmetry in the ordered phase.^61^ However, p-doping is known to inhibit spontaneous long-range ordering in InGaP.^63,64^ Therefore, one can reject the ordering hypothesis as the origin of the L1 band observed in our NWs.

According to the above considerations, we assign the L1 band to the LOPCM associated with the coupling of the GaP-like LO phonon (LO1) and the heavy hole plasma, the X-band plasma in the case of n-In_0.35_Ga_0.65_P. The LOPCM amplitude is expected to increase with increasing free carrier density until it reaches saturation due to the complete screening of the surface built-in electric field. The LO1 phonon recorded in the spectra arises from the surface depletion region, whose depth depends on the free carrier concentration. Therefore, the relative intensity of the LOPCM to the LO1 phonon mode is related to the free carrier concentration. This ratio is very high in the p^+^-InGaP tunnel diode, which accounts for a free hole concentration of around 1 × 10^20^ cm^–3^. This ratio decreases in the p-InGaP end of the top cell, which indicates a lower hole concentration there. The similar peak frequency of the LOPCM in both sectors is due to the combination of free hole concentration and plasma damping. In overdamped plasmas, one can have similar LOPCM frequencies for different combinations of free carrier concentration and plasma damping.^65^ It should be noted that the plasma damping increases with the doping concentration because the mobility is decreased and the band filling increases the nonparabolic character of the bands, with the concomitant increase of the hole effective mass.^66^

In order to get a better understanding of the nature of the L1 band, we modeled the LOPCMs in p-type In_*x*_Ga_1–*x*_P for a value of *x* = 0.25, corresponding to the composition estimated for the p^+^-InGaP segment of the tunnel diode. As mentioned above, the phonon spectrum of InGaP remains a matter of controversy. Most of the III–V alloys behave as two-mode systems, with two LO modes and two TO modes, corresponding to each of the binary constituents of the alloy. However, InGaP exhibits a modified two-mode behavior with two LO modes (LO1 and LO2) and one TO (TOM).^24,25^ The other TO mode (TOm) behaves as an impurity mode connecting the local mode of Ga in InP with the resonant mode of In in GaP. If one assumes a two-mode behavior, we have to take LO1 and TOm associated with the GaP sublattice and LO2 and TOM associated with the InP sublattice. However, calculations of the phonon density of states (DOS), establish the participation of both In and Ga in the TOM mode, therefore, the TO modes of InP and GaP merge into the TOM alloy mode.^30^

**Figure 10:**
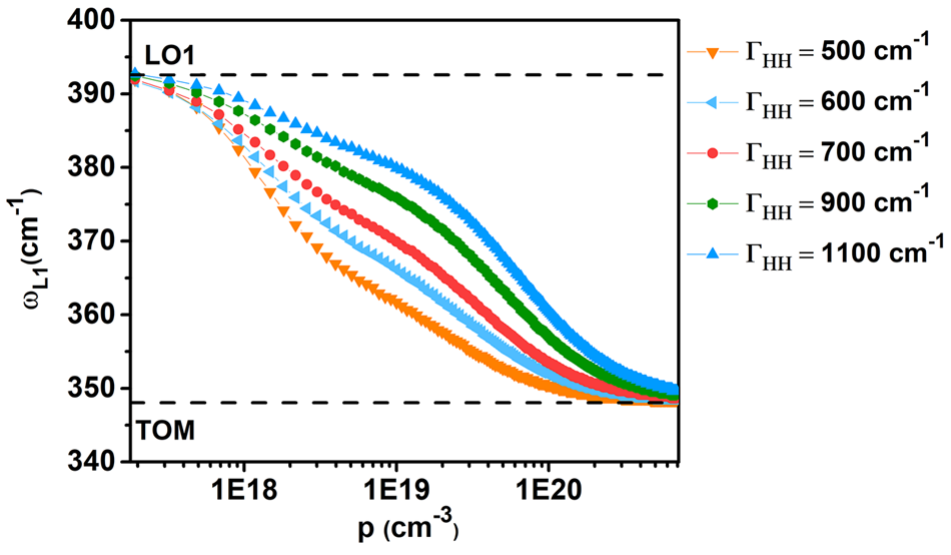
Calculated L1 peak frequency vs hole concentration for different damping constants.

Dielectric modeling of LOPCMs was developed by Hon and Faust for binary semiconductors.^67^ Later on, it was applied to p-type GaAs.^54,55^ Cuscó et al. extended the Hon-Faust model to n-type ternary alloys.^68^ Further development led to the use of the model for p-type-doped InGaAs.^69^ The dielectric function used for modeling the LO phonon–hole plasma coupling includes the phonon and free carrier (holes) contributions using the Lindhard–Mermin formalism. Only the heavy hole (HH) intraband contributions to the susceptibility are considered.^67^ The initial calculations, taking a two-mode behavior into account, including LO1, LO2, TOM, and TOm, give unrealistic results. Under such an assumption, the LOPCM frequencies were underestimated, especially in the low carrier concentration region. On the other hand, high plasma damping must be considered to account for the rather insensitive LOPCM frequency shift in the concentration range of the tunnel diode. This is relevant in our system since the high effective mass of the heavy hole band, *m*_HH_ = (0.6 + 0.19 (1 – *x*))*m*_e_ ≈ 0.74*m*_e_ (*m*_e_ is the electron mass) for *x* = 0.25, limits the carrier mobility resulting in an overdamped hole plasma. Moreover, one has to consider that the TOM phonon involves the participation of In and Ga atoms,^30^ but for *x* = 0.25, it must be largely contributed by the Ga atoms. This, together with the dominant LO1 mode over the LO2 mode, suggests that the phonon–plasmon coupling can be treated in the frame of an alloy with one mode behavior.^29^ The best fitting was achieved by using only one mode in the Lindhard–Mermin formalism by considering the LO1 and TOM modes of the alloy (SI 3). Within this framework, the calculations gave a good accuracy, which could not be reached in the two-mode approach. The parameters used in our calculations are listed in Table SI1. The frequencies of the LOPC mode as a function of the free hole density for different damping constants are plotted in Figure 10. The calculations confirmed the one-mode behavior for the phonon–plasmon coupling in agreement with the results obtained in n-doped InGaP by Lee et al.^29^

**Figure 11:**
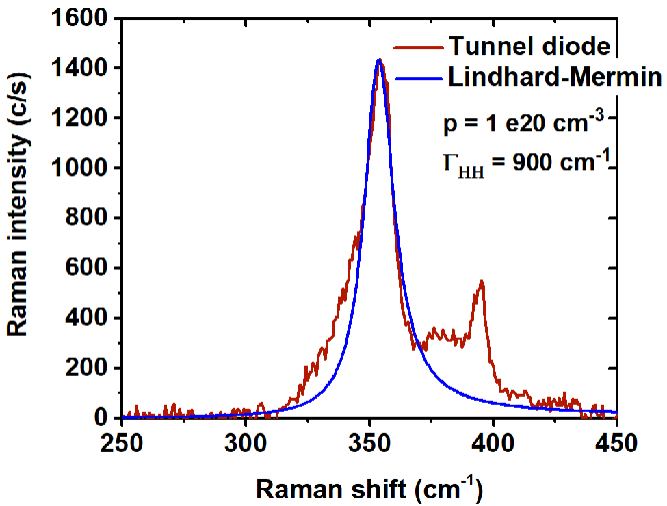
Raman spectrum of the p^+^-InGap side of the tunnel diode and the fitting of the L1 band using the Lindhard–Mermin formalism for the dielectric susceptibility.

Even if the L1 peak frequency does not determine the free carrier concentration, as the same peak frequency can be recorded with different pairs of free carrier concentrations and plasmon damping constants, a fitting of the L1 band in the full Raman spectra permits us to obtain the free hole concentration and plasma damping. The best fit for the p^+^InGaP side of the tunnel diode was achieved for a free hole concentration of ≈1 × 10^20^ cm^–3^, and plasma damping constant of 900 cm^–1^, Figure 11.

In order to facilitate carrier tunneling, the tunnel diode must have a narrow space charge region, which is achieved by degenerate doping. The Raman spectra acquired in the tunnel diode do not show the presence of intrinsic InGaP, or compositionally graded InGaP, which evidence that the carrier depletion thickness is very narrow as one cannot resolve this region in the Raman spectrum. Note that the HJ optical resonances are very sensitive to the dielectric function changes; therefore, an increase of the carrier depletion thickness would be revealed by the Raman spectrum because of the resonance at the HJ. Electrical measurements on InP NWs are plagued by poor ohmic contacts to the p-side, which renders the estimation of the free carrier concentration difficult. However, the *I*–*V* plots of axially heterostructured p^+^-In_*x*_Ga_1–*x*_P/n^+^-InP nanowires grown under similar conditions as those analyzed herein showed clear negative differential resistance (NDR),^21,51^ revealing the existence of the tunneling effect accounting for degenerate n and p doping, corresponding to free carrier concentrations in the neighborhood of the 10^20^ cm^–3^, as estimated by Raman spectroscopy.

## Conclusion

We reported on Raman spectra recorded along photovoltaic tandem junction InP/GaInP NWs. The spectra reveal the characteristics of the different segments in the NWs. The InP sectors give a weak Raman signal (at least 1 order of magnitude with respect to the other regions of the NW), more in particular, the n^+^-InP side of the tunnel diode appears to be Raman blind. The interaction between the laser beam and the NW results in an optical resonance at the p^+^-InGaP side of the tunnel junction, while the electromagnetic field appears to be ejected from the n^+^-InP side. The Raman spectrum of the tunnel diode presents a strong mode that behaves as an LOPCM that has not been previously reported. Its strong intensity is related to the electromagnetic resonance at the heterojunction. The composition of InGaP is affected by doping, in particular, Zn is shown to reduce the incorporation of In, resulting in the p-doped sectors being gallium-rich. S-doping does not produce such an effect.

The Lindhard–Mermin formalism for the dielectric function was used for both simulation of the laser/NW interaction and modeling of the LOPCMs. The experimental results and the modeling of the LOPCMs support the claim that phonon–plasmon coupled modes follow a one-mode behavior.

## Materials and Methods

To synthesize the tandem junction NW arrays, we defined a hexagonal pattern optimized for light absorption of Au discs with a pitch of 500 nm on 2 in. InP (111)B n-type wafers using displacement Talbot lithography (TDL), metal evaporation, and lift-off, and the wafers were diced into 9 × 11 mm sized substrates. The axially heterostructured NWs have a tandem junction structure, consisting of an InP (n-i-p) bottom cell, a tunnel diode (p^+^InGaP/n^+^InP), and an InGaP (n-i-p) top cell. We used a laminar flow Aixtron 200/4 MOVPE reactor with a total flow of 13 l × min^–1^ using hydrogen as a carrier gas at a reduced pressure of 100 mbar. To preserve the periodic pattern of catalytic metal particles for synthesis, we used a low-temperature prenucleation step, in which we supplied trimethylindium (TMIn) and phosphine (PH_3_) with molar fractions of χTMIn = 8.91 × 10^–5^ and χPH_3_ = 6.92 × 10^–3^ at 280 °C. Subsequently, we closed the TMIn line to the reactor, increased the PH_3_ flow to χPH_3_ = 3.46 × 10^–2^ and annealed the samples at 550 °C to desorb any surface oxide from the surface. Afterward, the temperature was reduced to the growth temperature of 440 °C and the PH_3_ flow to χPH_3_ = 6.92 × 10^–3^. Then, TMIn was reintroduced (χTMIn = 5.94 × 10^–5^) to initiate NW growth. At the same time, and for the rest of the NW growth, we used hydrogen chloride (χHCl = 1.23 × 10^–4^) to prevent radial growth.^12^ We used hydrogen sulfide (H2S) as n-dopant (χH_2_S = 3.11 × 10^–5^) and diethylzinc (DEZn) as p-dopant. As nominally intrinsic InP shows n-type behavior, we used a low DEZn molar fraction (χDEZn = 9.51 × 10^–8^) for compensation doping of the middle segment of the n-i-p junction and afterward increased it to χDEZn = 1.11 × 10^–5^ for growth of the p-segment. To continue with the GaInP selective barrier, we added triethylgallium (TEGa) with a molar fraction of χTEGa = 9.25 × 10^–5^. To form the Esaki tunnel diode, we turned off TEGa and DEZn and opened H_2_S. To avoid kinking, we reduced the flows of H_2_S, TMIn, and TEGa for the top-cell (χTMIn = 2.97 × 10^–5^, χTEGa = 4.48 × 10^–5^, χH_2_S = 3.02 × 10^–6^). For more details, we refer to Hrachowina et al.^70^

The similar atomic size of S and P favors S entering into the P sites resulting in shallow donor levels, 5.7 meV in InP, and 107 meV in GaP.^71^ The nominal In material composition in InGaP was ≈0.35, which is close to the direct to indirect bandgap crossover of the InGaP alloy.

The NWs were deposited on a Si substrate coated with a gold layer, which permits enhancement of the Raman signal, but also allows heat dissipation avoiding the NW overheating by the laser beam; the laser power was kept below the heating threshold.

Raman spectra were acquired using a LabRAM SOLEIL spectrometer (Horiba; Kyoto, Japan) equipped with a Symphony CCD detector. The exciting beam was generated by a solid-state laser (λ = 532 nm) with a standard power density of ≈100 KW cm^–2^. Under these excitation conditions, the temperature of the NW was not increased as the peak positions did not change when varying the power excitation around this value. The slit aperture was 100 μm ensuring a spectral resolution better than 1 cm^–1^. The diameter of the laser beam at the focus was ≈0.7μm according to the Abbe formula.

The laser beam was scanned along the NW axis in steps of 200 nm. The laser polarization was parallel to the NW axis to obtain the maximum Raman signal, while the scattered light was not analyzed (*z*(*x*-)-*z* scattering configuration). Raman spectra of In_*x*_Ga_1–*x*_P epitaxial layers (*x* ≈ 0.5) lattice matched to GaAs,^72^ both undoped and p-doped, were recorded as references for the analysis of the Raman spectra of NWs.

## Supporting Information Available

S1: Raman spectroscopy, S2: HRTEM, S3: LOPCM modeling, S4: Energy-dispersive X-ray spectroscopy (PDF)
S5: Animated representation of the electromagnetic field distribution when scanning the laser beam along the tunnel diode (MP4)
