# Supplementary material for: Composition, Optical Resonances, and Doping of InP/InGaP Nanowires for Tandem Solar Cells: a Micro-Raman Analysis
Source: ACS Nano. 2024 Mar 27;18(14):10113–23. doi: 10.1021/acsnano.3c12973 (PMC11008355; doi:10.1021/acsnano.3c12973)
Supplement: Supplementary file 1 — nn3c12973_si_001.pdf [file nn3c12973_si_001.pdf]

Supporting Information: Composition, optical resonances, and doping of InP/InGaP nanowires for tandem solar cells: a micro-Raman analysis

Irene Mediavilla[1], José Luis Pura[1,2], Vanessa Giselle Hinojosa[1], Beatriz Galiana[3], Lukas Hrachowina[4], Magnus T. Borgström[4], Juan Jimenez[1*]

[1]GdS Optronlab, Ed. LUCIA, University of Valladolid, Paseo de Belén 19, 47011 Valladolid, Spain

[2]Instituto de Estructura de la Materia (IEM-CSIC), Consejo Superior de Investigaciones Científicas, Serrano 121, 28006 Madrid, Spain

[3]Universidad Carlos III de Madrid, Physics Department, Av. Universidad 40, Leganes 28911, Spain

[4]NanoLund and Divison of Solid StatePhysics, Lund University, Box 118, 221 00 Lund, Sweden

---

S1. Raman spectroscopy

This section presents the Raman results obtained for several NWs within the same growth run as the 10 μm long NW presented in the main text. This is only a sample of the NWs studied.

Fig. SI.1 illustrates the Raman spectra obtained from nanowires of different lengths, demonstrating an overall similarity, although with some distinctions attributed to the different growth rate for different NW lengths.

When the laser beam enters the tunnel diode, the Raman signal arising from the p^{+}- InGaP side of the tunnel diode is enhanced. All the NWs exhibit the L1 mode 355 cm^{-1}, along

with the three phonon modes of InGaP: LO1 ($\sim$ 392 cm^{-1}), LO2 ($\sim$ 378 cm^{-1}) and, TOM ($\sim$ 345 cm^{-1}). The presence of this last mode is evident from the asymmetrical shape of the low frequency side of L1 band.

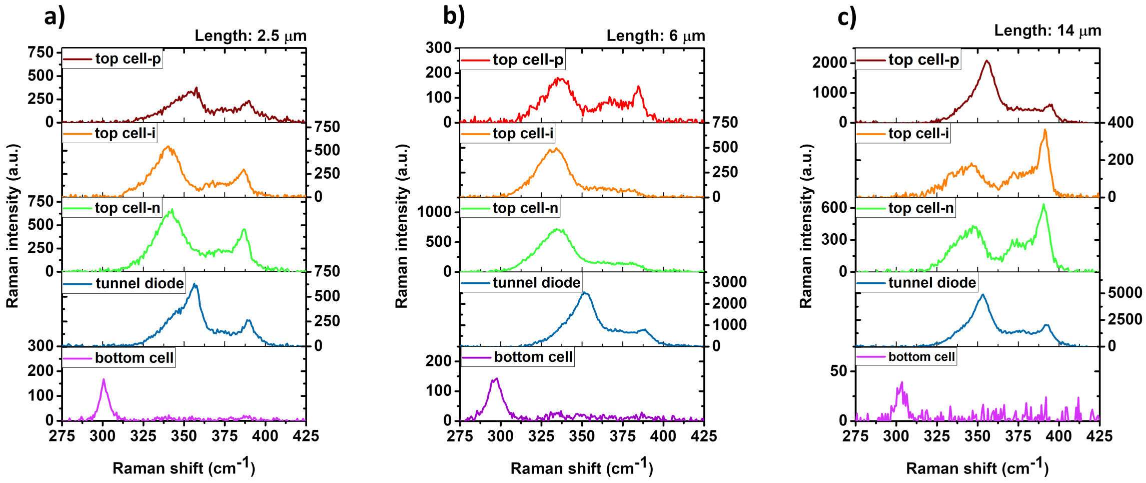

Figure SI.1: Selected Raman spectra representative of each sector of the NW for different length NW **a)** 2.5 μm, **b)** 6 μm, and **c)** 14 μm.

In the 2.5 μm long NW, **Fig. SI.1a**, we can observe that in the spectrum associated with the tunnel junction, the TO mode of InP from the bottom cell can also be identified. This is because, being a shorter nanowire, the size of the laser spot plays a crucial role in distinguishing the various sectors of the NW. In short NWs, the laser spot shares several sectors of the NW in a single measurement.

It is significantly easier to distinguish the signal coming from the different NW sectors in long NWs. For this reason, a 10 μm long NW was chosen for the full analysis presented in the main text, as it allows us for a more accurate study of the different NW sectors.

Finally, it is worth noting the remarkable difference observed in the Raman signal from the NW tip when it does not retain (**Fig.SI.1a** and **Fig.SI.1b**) and retains (**Fig.SI.1c**) the gold droplet. The presence of the gold droplet substantially enhances the L1 mode, as it induces an electromagnetic resonance.

S2. High-resolution transmission electron microscopy

High-resolution transmission electron microscopy (HRTEM) measurements were carried out in a JEOL JEM 3000F. The nanowires were deposited on a carbon grid for TEM observation.

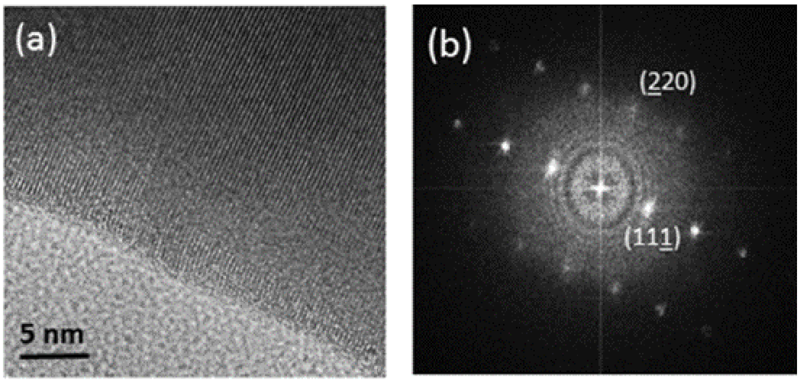

Figure SI.2: a) HRTEM image of the InP bottom cell, **b)** Fast Fourier Transform (FFT) of the HRTEM a).

Fig.SI.2a and **Fig.SI.2b** show a high-resolution TEM image and the corresponding fast Fourier transform (FFT), which simulates the experimental electron diffraction pattern (EDP), of a region of the InP bottom cell of the nanowire. The diffraction spots were indexed as (111) and (220) reflections of the ZB InP phase.

Fig.SI.3a and **Fig.SI.3b** show a high-resolution TEM image and the corresponding FFT of the interface of InP/InGaP axially heterostructured NW which corresponds to the InGaP/InP tunnel junction.

The FFT shown in **Fig.SI.3b** reveals the existence of two ZB structures that are aligned along the (111) axis. The spots marked in red correspond to the [011]-InGaP planes, and those marked in blue to [011]-InP. The inverted images for both set of planes are shown in **Fig.SI.3c** and **Fig.SI.3d**, where the InGaP tunnel junction region (**Fig.SI.3c**) and the InP region (**Fig.SI.3d**) are well defined.

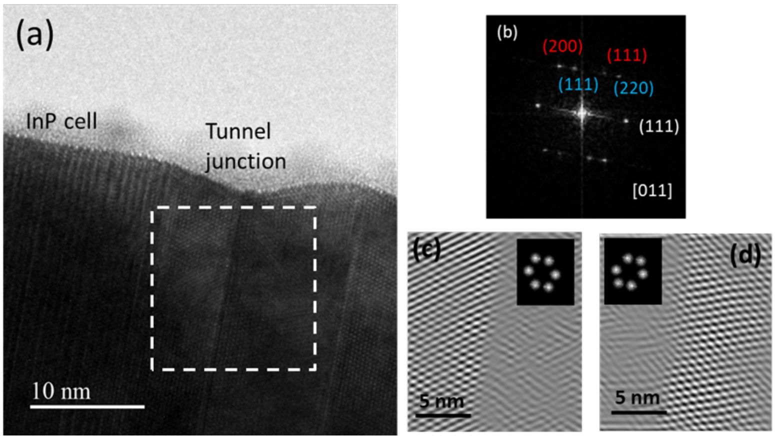

Figure SI.3: a) HRTEM of the tunnel junction between InP and InGaP subcells, **b)** FFT of the TEM image shown in a), **c)** inverted image extracted from the indexed planes marked in red in figure b) that corresponds to [011]-InGaP and, **d)** inverted image extracted from the indexed planes marked in blue in figure b) that corresponds to [011]-InP.

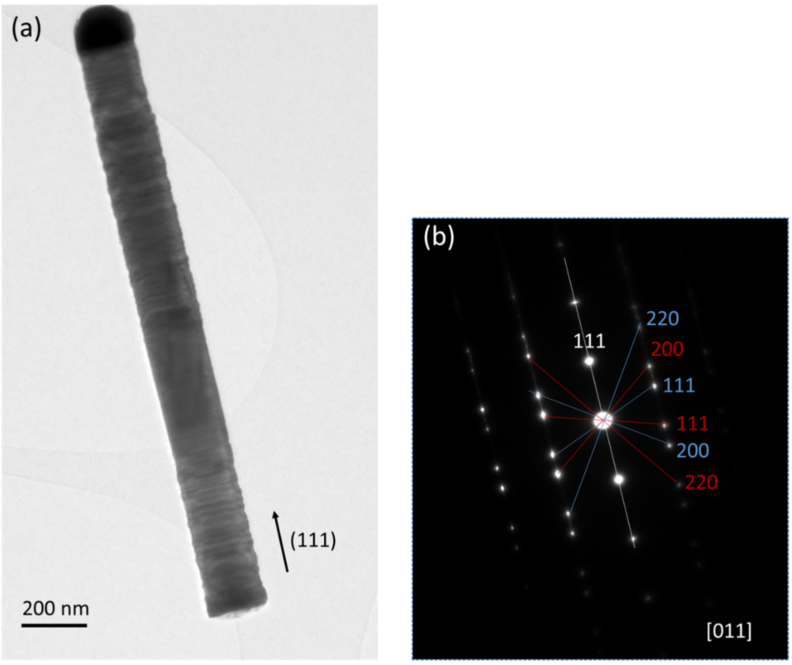

Figure SI.4: a) TEM image of a InP/InGaP nanowire, **b)** electron diffraction patter (EDP) of the complete nanowire where two ZB structures in the [011] zone axis are identified. The planes marked in red belongs to ZB [011]-InGaP and the planes marked in blue to ZB [011]-InP.

Fig.SI.4a and **Fig.SI.4b** display a TEM image and the corresponding experimental electron diffraction pattern of a InP/InGaP nanowire. The diffraction spots were indexed showing the existence of two ZB structures. According to the previous HRTEM (**Fig.SI.3**) it can be identified ZB [011]-InGaP planes in red and ZB [011]-InP planes in blue. This result confirms the ZB structure in both InGaP and InP over the complete nanowire.

S3. LOPCM modeling

The Raman spectra are analysed using a dielectric model based on the Lindhard-Mermin susceptibility taking into account only the heavy holes (HH) contribution. The main parameters used in our calculations are listed in **Table SI.1**.

Table SI.1 List of parameters used in the LOPCM model.

Symbol	Description	Value
m^*_{HH}	Heavy-hole effective mass	0.74 me
C	Faust-Henry coefficient	-0.5125
Γ_I	Ionic damping constant	7 cm^{-1}
T	Temperature	300 K
ε_∞	High-frequency dielectric constant	9.235
ω_{TO}	TO-phonon wavenumber GaP	348.2 cm^{-1}
ω_{LO}	LO-phonon wavenumber GaP	394.1 cm^{-1}

The differential Raman cross section for the LOPCM's of a doped two-mode ternary alloy $A_xB_{1-x}C$ can be expressed as:

$$\frac{\partial^2\sigma}{\partial\omega\partial\Omega} \propto [n(\omega)+1]\,\mathrm{Im}\left\{\frac{-1}{\varepsilon(\omega,x)}\left[\frac{1}{4\pi}+2\sum_{i=1}^{2}\frac{A_i}{\varepsilon_{\infty,i}}\chi_i(\omega,x) -4\pi\left(\frac{A_1}{\varepsilon_{\infty,1}}-\frac{A_2}{\varepsilon_{\infty,2}}\right)^2\chi_1(\omega,x)\chi_2(\omega,x) -\left(1+\frac{4\pi\chi_h(\omega)}{\varepsilon_\infty(x)}\right)\varepsilon_\infty(x)\sum_{i=1}^{2}\left(\frac{A_i}{\varepsilon_{\infty,i}}\right)^2\chi_i(\omega,x)\right]\right\} \quad (1)$$

Here $n(\omega)$ is the Bose distribution function, $\varepsilon_\infty(x) = x\,\varepsilon_{\infty,A}+(1-x)\,\varepsilon_{\infty,B}$ is the average high-frequency dielectric function, and the dielectric function of the alloy is given by:

$$\varepsilon(\omega,x) = \varepsilon_\infty(x)+4\pi\sum_{i=A,B}\chi_i(\omega,x)+4\pi\chi_e(\omega) \quad (2)$$

where ε_∞ is the high frequency dielectric constant, $\chi_h(\omega)$ is the electric susceptibility of the free-charge plasma, and $\chi_i(\omega,x)$ is the ionic susceptibility contributions from each sublattice given by:

$$\chi_i(\omega,x) = x_i\,\frac{\varepsilon_{\infty,i}}{4\pi}\,\frac{(\omega^0_{LO,i})^2-(\omega^0_{TO,i})^2}{\omega^0_{TO,i}-\omega^2-i\Gamma_i\omega} \quad (3)$$

$\omega^0_{TO,i}$ and $\omega^0_{LO,i}$, with $i = A,B$, are the TO and LO phonon frequencies of the pure end-member compounds, $\omega_{TO,i}$ is the TO phonon frequency of the alloy i sublattice, and Γ_i is the phonon damping parameter.

However, as the results suggest that the phonon plasmon coupling can be treated as one mode behavior, we only have taken into account the GaP- sublattice in the calculations, so

the cross term in Eq.(1) has been eliminated. Thus, we have used the one mode Lindhard-Mermin formalism.

The constants A_i introduced in Eq.(1) are defined as:

$$A_i = C_i^0 \frac{\omega_{TO,i}^2}{(\omega_{LO,i}^0)^2 - (\omega_{TO,i}^0)^2} \quad (4)$$

with C_i^0 the Faust-Henry coefficient for the pure end-member compound.

We used the Lindhard-Mermin model to calculate the free hole contributions to the susceptibility (χ_h):

$$\chi_h(q, \omega + i\Gamma_h) = \frac{(1 + i\Gamma_h/\omega)\chi_h^L(q, \omega + i\Gamma_h)}{1 + i\Gamma_h \chi_h^L(q, \omega + i\Gamma_h)/(\omega\chi_h^L(q, 0))} \quad (5)$$

where the heavy-hole intraband contributions to the Lindhard susceptibility was given by:

$$\chi_h = \frac{e^2}{2\pi^3 q^2} \int f_h(E_F^h, T, k) \frac{E_h(q+k) - E_h(k)}{[E_h(q+k) - E_h(k)]^2 - (\hbar\omega)^2} d^3k \quad (6)$$

where $f_h(E_F^h, T, k)$ is the Fermi distribution function for a hole plasma with Fermi energy E_F at temperature T, and $E(k)$ is the energy dispersion of the alloy conduction band.

S4. Energy-dispersive X-ray spectroscopy (EDX)

The chemical composition analysis was carried out using a Scanning Electron Microscope (FEI-QUANTA 200FEG, Hillsboro, OR, USA) with Energy Dispersive Spectrometry. The system has a Schottky's Filament Field Emission Cannon, and the results were

achieved at 10 kV. An EDAX Genesis micro-probe (Mahwah, NJ, USA) was used for elemental microanalysis.

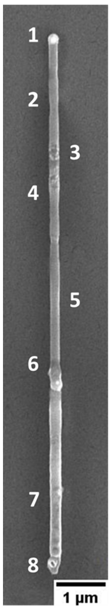

Point	Element	at%	
1	In	0.61	Au droplet
	Ga	0.39	
2	In	0.36	p-InGaP
	Ga	0.64	
3	In	0.38	i-InGaP
	Ga	0.62	
4	In	0.38	
	Ga	0.62	
5	In	0.37	n-InGaP
	Ga	0.63	
6	In	0.27	Tunnel diode
	Ga	0.73	
7	In	1	InP cell
	Ga	--	
8	In	1	
	Ga	--	

Figure SI.5: SEM image of the nanowire with the numbered points where the EDX measurements have been carried out, and the concentration of In and Ga in each point measured.
